# Suture fixation of migrated septal occluder device to prevent further migration: a simple surgical technique

**DOI:** 10.1186/1749-8090-8-10

**Published:** 2013-01-16

**Authors:** Prashant N Mohite, Sachin A Kuthe, Shrirang D Ranade, Pravin P Kulkarni, Anton Sabashnikov, Aron F Popov

**Affiliations:** 1 Department of Cardiothoracic and Vascular surgery, Postgraduation Institute of Medical Research and Education, Sector- 12, Chandigarh 160012, India; 2 Department of Cardiothoracic Transplantation and Mechanical Support, Royal Brompton and Harefield NHS Trust, Hill End Road, UB9 6JH, London, UK; 3 Department of Thoracic Cardiovascular Surgery, University of Göttingen, Robert-Koch- Strasse 40, 37099, Göttingen, Germany

**Keywords:** Atrial septal defect, Septal occlude device, Transcatheter closure, Migration of occlude device

## Abstract

As the use of percutaneous intervention is increasing for the closure of the atrial septal defect, the procedure related complications are also on rise, migration of the device being most common. The migrated devices with failed percutaneous retrieval must be removed surgically under cardiopulmonary bypass. During establishment of cardiopulmonary bypass, the handling of heart may cause further migration of the device into other chambers of heart which leads to difficulty in finding and retrieval of the device. The authors propose a simple and unique technique to prevent further migration of the septal occluder device.

## Introduction

The transcatheter closure of the atrial septal defect (ASD) has been proposed as an alternative to surgical therapy for the ostium secundum ASD. The transcatheter closure ASDs was first described by King and Mills in 1976 [1]. It is considered an effective and safe procedure, and although rare but potentially fatal complications have been reported. Migration of the septal occluder devices is a common complication of the procedure [2]. The migrated devices with failed percutaneous retrieval must be removed surgically under cardiopulmonary bypass.

## Methods

**Figure 1:**
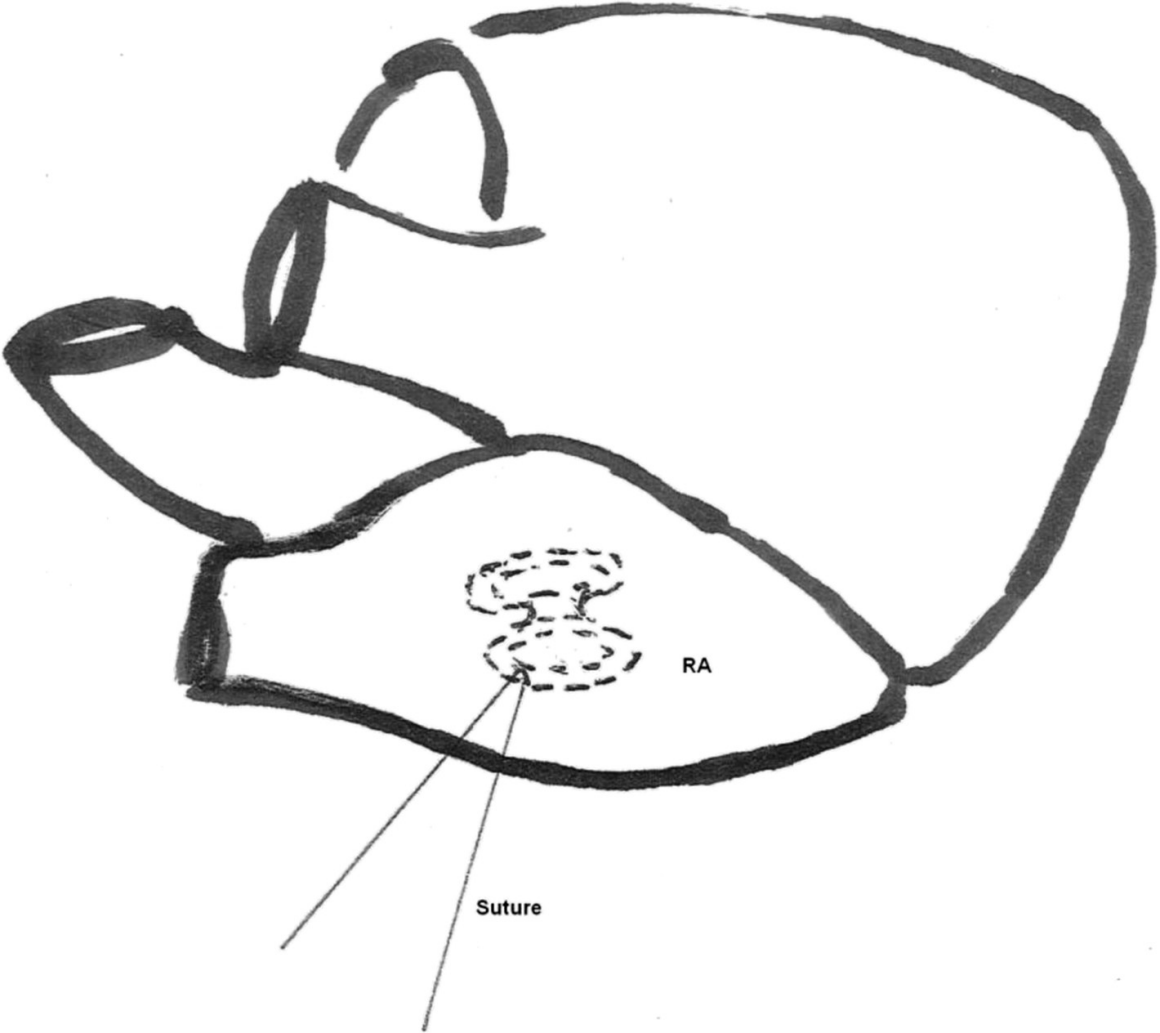
Suture passing through the occlude device from outside the right atrium.

As soon as the pericardium is opened, the migrated device is palpated. Pre-operative fluoroscopy or simply transthoracic echocardiography locates the migrated device. As the device is palpated, it is held in the pair of forceps along with the wall of the chamber, mostly right atrium. A 3-0 polypropelene suture is entered through the wall of the chamber passing through the migrated device and knot is tied (Figure 1). Aorto- bicaval cannulation is then done and cardiopulmonary bypass is established. Heart arrested with topical hypothermia and high potassium cardioplegia. Right atrium is incised obliquely and the device is found stuck to the atrial wall with the polypropelene suture. The device is retrieved after cutting the suture and atrial septal defect is closed directly or with patch.

## Discussion

Recently, percutaneous transcatheter procedures emerged as therapeutic alternatives for closure of both atrial septal defects and patent foramen ovale. Percutaneous closure of ASDs is an excellent therapeutic option for the appropriate patient, yielding comparable results to surgery with minimal morbidity and complications. Unfortunately, however, such percutaneous procedures may require surgical intervention for early or late complications. Percutaneous ASD closure has proven to be reliable, safe, and effective [3]. The reported success rate of these devices in closing ASD is as high as 98% [4]. Periprocedural complications include air embolism, device migration, cardiac tamponade, device erosion, atrial arrhythmias, and those related to vascular access [2]. Moreover, device related complications include device migration, device fracture, device thrombosis, and device erosion. Device embolization occurs in about 0.55% of cases, regardless of ASD size, device size, or the physician’s expertise [5].

There are several reasons for the acute failure of these devices. The most important is suboptimal patient and/ or device selection [6,7]. Other suggested mechanisms of acute failure are device related failure, inadequate experience, poor defect rim to hold the device [4-6], and tearing of the interatrial septum as a result of catheter and device manipulations [7]. It has been reported by Balbi M et al. that percutaneous retrieval was successful in 50% of the cases of ASO embolization that have been reported in the literature [8].

Once the device is migrated, it may slip into right atrium, right ventricle to pulmonary artery or into left atrium, left ventricle to aorta. Surgical intervention becomes mandatory when the migrated device could not be retrieved by percutaneous catheter intervention [9]. Urgent surgery and device retrieval is necessary when device gets migrated into heart chambers and causes arrhythmia or hemodynamic compromise.

The migrated septal occluder device may be further migrated due to manipulation of the heart while putting patient on cardiopulmonary bypass. Authors suggest palpating the migrated device gently and holding it with the pair of forceps. The device is then sutured from outside to the wall of the chamber containing it with fine polypropelene suture. Once the device is thus secured to the chamber wall, procedure can be progressed without hesitation and fear of further migration of device. This technique which does not consume much time is useful in occluder device migration in any patient irrespective of age or urgency of the procedure.

## Competing interests

The authors declare that they have no competing interests.

## Authors’ contribution

PNM: Concept, wrote the manuscript; SDR, PPK and AS: gave important comments; AFP: made critical revision of the article and drafted the manuscript. All authors read and approved the final manuscript.
